# A Novel Class of HIV-1 Antiviral Agents Targeting HIV via a SUMOylation-Dependent Mechanism

**DOI:** 10.1038/srep17808

**Published:** 2015-12-08

**Authors:** Ikenna G. Madu, Shirley Li, Baozong Li, Haitang Li, Tammy Chang, Yi-Jia Li, Ramir Vega, John Rossi, Jiing-Kuan Yee, John Zaia, Yuan Chen

**Affiliations:** 1 Department of Molecular Medicine, 1500 East Duarte Road, CA 91010.; 2 Department of Virology, 1500 East Duarte Road, CA 91010.; 3 Department of Molecular and Cellular Biology, Beckman Research Institute of the City of Hope, 1500 East Duarte Road, Duarte, CA 91010

## Abstract

We have recently identified a chemotype of small ubiquitin-like modifier (SUMO)-specific protease (SENP) inhibitors. Prior to the discovery of their SENP inhibitory activity, these compounds were found to inhibit HIV replication, but with an unknown mechanism. In this study, we investigated the mechanism of how these compounds inhibit HIV-1. We found that they do not affect HIV-1 viral production, but significantly inhibited the infectivity of the virus. Interestingly, virions produced from cells treated with these compounds could gain entry and carry out reverse transcription, but could not efficiently integrate into the host genome. This phenotype is different from the virus produced from cells treated with the class of anti-HIV-1 agents that inhibit HIV protease. Upon removal of the SUMO modification sites in the HIV-1 integrase, the compound no longer alters viral infectivity, indicating that the effect is related to SUMOylation of the HIV integrase. This study identifies a novel mechanism for inhibiting HIV-1 integration and a new class of small molecules that inhibits HIV-1 via such mechanism that may contribute a new strategy for cure of HIV-1 by inhibiting the production of infectious virions upon activation from latency.

New HIV-1 infections continue to occur, and despite the success of antiretroviral therapy (ART), resistance to current therapies is a major challenge. In addition, patients that undergo successful ART treatment still experience HIV-1-infection-related co-morbidities, such as increased incidence of cardiovascular, bone and cognitive disorders^1^. HIV-1 persistence is, at least in part, due to latently infected CD4^+^ T-cells that can be activated, leading to viral production^2^. Low-level viral replication may also occur when patients are treated with ART, resulting in continuous infection of new host cells^3^,^4^. Studies of HIV-1-host interaction are key to identifying effective strategies to achieve a complete cure by eliminating all latently infected cells, and/or a functional cure by enhancing the host ability to control persistent viral infection.

Recently, we identified a chemotype of small molecule inhibitors of SENPs, which are small ubiquitin-like modifiers (SUMO)-specific proteases, and we called them SUMO-protease inhibitor (SPI)^5^. Post-translational modifications by SUMO regulate diverse cellular functions and do not directly target proteins for degradation^6^,^7^. SUMOylation is initiated and removed by the activities of SUMO-specific proteases (SENPs)^8^. There are 6 SENPs, organized into three families based on sequence similarity: SENP1 and 2 that catalyze maturation of SUMO precursors and removal of SUMO-1 and SUMO-2/3 conjugates; SENP3 and 5 that preferentially remove SUMO-2/3 conjugates; and SENP6 and 7 that appear to be mainly involved in editing poly-SUMO-2/3 chains^9^,^10^. Recently, another de-SUMOylase was discovered but does not share sequence similarity with the SENPs^11^. Prior to the discovery of SPI as SENP inhibitors^12^, two members of what we now term a “SPI class”, SPI-01 (PubChem ID: NSC5068) and SPI-02 (PubChem ID: NSC16224)^12^, were found in a high-throughput drug screen to have anti-HIV-1 activity^13^. The CC_50_ and EC_50_ of SPI-01, measured by NCI during their anti-HIV screen were 115 μM and 63 μM, respectively for the CEM-SS cell line. However, the mechanisms of their anti-HIV effects were unknown.

In this study, we now investigated by what mechanism these SPI agents inhibit HIV-1 infection. We found that these inhibitors neither inhibited the infection of normal HIV-1 into host cells nor the first cycle of production of HIV-1 virions. However, the HIV-1 virions produced from cells treated with the inhibitors failed to integrate into the host genome, although they could carry out cellular entry and reverse transcription. These characteristics were associated with packaging of SUMO modified integrase into the virus. In addition, removal of the SUMO modification sites in the HIV-1 integrase also removed the inhibitory effects of the compounds. Taken together, this study has revealed a novel mode of action for inhibiting HIV-1 integration into the host genome as well as a chemotype of small molecules that confer such effect.

## Results

### Minimal effects of SENP inhibitor on cell viability and virus production

**Figure 1:**
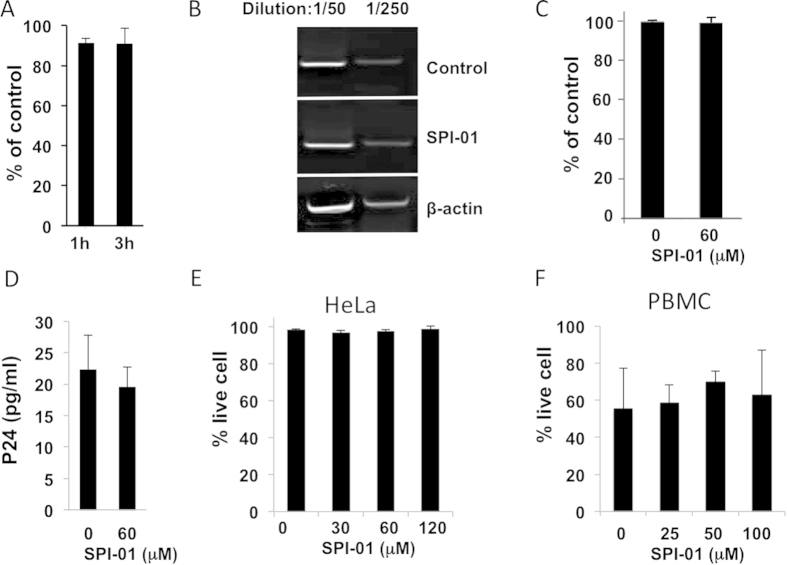
SPI-01 inhibited HIV-1 replication but did not inhibit production of virus. (**A**) Expression of luciferase as percent of untreated control following transduction of HeLa cells with VSV-G pseudotyped pNL43LUCR^-^E^-^ showing no significant inhibition by treatment of the cells with 60 μM SPI-01 for either 1 or 3 h. (**B**) SPI did not significantly block viral integration. HeLa cells treated overnight with 120 μM SPI-01, and transduced with DNase-treated VSV-G pseudotyped virus supernatant. To detect integration products, 48 h post infection the isolated total cellular DNA was analyzed by nested PCR using Alu- and viral-specific primers. β-actin primers were used for normalization. PCR products were diluted by 50 or 250 folds and visualized by ethidium bromide stained agarose gel showing the DNA bands after PCR. (**C**) Tat activation of HIV-1 LTR-luciferase after treatment with SPI-01. 293T cells were cotransfected with HIV-1-LTR-Luc and Tat expression plasmid and treated with 60 μM SPI-01. After transfection (24 h), luciferase activity was assessed, normalized against Renilla luciferase, and compared to the control to determine % activity. (**D**) VSV-G pseudotyped pNL43LUCR^-^E^-^ viral production (assessed by p24 levels) in HeLa cells treated with 60 μM SPI-01 or mock treated. No significant reduction was observed. (**E**) Representative data showing that SPI-01 confers minimal toxicity. HeLa cells were treated with indicated concentrations of SPI-01 for 72 h. Dead cells were assayed by trypan blue staining. (**F**) CD8-depleted PBMCs, maintained in TCM 1000 ActiCyte media (BioE), challenged with HIV-1 NL4-3 (MOI of 0.01) were treated with SPI-01 at the indicated concentrations for 12 days. Percent of live cells were determined by the Live/Dead® Fixable Dead Cell Stain Kits (Life technologies).

SPI-01 was used as the representative of this chemotype to investigate the mechanism of inhibiting HIV. Various steps of viral replication were initially investigated using a VSV-G pseudotyped HIV-1 vector, pNL43LUCR^-^E^-^, which serves as a single cycle reporter virus having a luciferase gene inserted in the *NEF* position and frameshift mutations in the *ENV* and *VPR* genes^14^. Expression of luciferase following transduction of HeLa, CEM-CCR5 and MT2 cells with VSV-G pseudotyped pNL43LUCR^-^E^-^ was not significantly inhibited by treatment with SPI-01 (p > 0.5, Fig. 1A), regardless of how long the virus or cells were pre-incubated with the inhibitor. In addition, SPI-01 did not affect integration as shown by the detection of properly integrated viral genome at the same level as mock treated cells (Fig. 1B)^15^. Furthermore, SPI-01 did not alter transcription activation as determined by Tat activation of HIV-1 LTR-luciferase (Fig. 1C) using the method described previously^16^. SPI-01 did not inhibit secretion of virus from cells transfected with the viral plasmids, as shown by p24 ELISA measurement (Fig. 1D). Consistent with the reported CC_50_ of 115 μM for the CEM-SS cells, when used at concentrations up to 120 μM, SPI-01 was not toxic to any tested cell lines (CEM-CCR5, MT2, HeLa, 293T and human peripheral blood mononuclear cells [PBMCs]), as determined by cell viability assay (representative data shown in Fig. 1E,F). Taken together, these results indicate that SPI-01 did not inhibit HIV-1 entry, integration or transcription activation.

### Inhibition of HIV-1 infectivity

**Figure 2:**
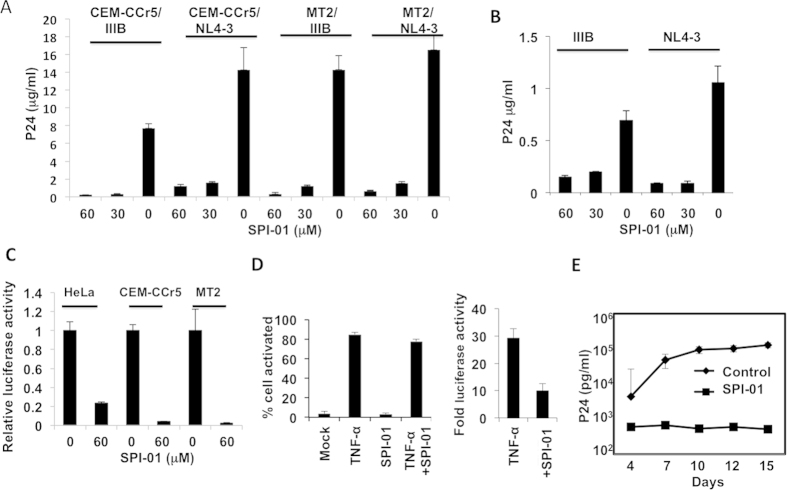
SPI-01 significantly reduces HIV-1 infectivity. (**A**) SPI-01 reduced, in a dose dependent manner, the infectivity of both HIV-1 NL4-3 and IIIB strains grown in CEM-CCR5 and MT2 cells. Cells were infected with the viruses for 16 h, and then excess virus and SPI-01 was washed away. Then cells were cultured for 7 days in media without the inhibitor, after which supernatants were collected and p24 levels measured by ELISA. (**B**) HIV-1 NL4-3 or IIIB strain obtained from the media after 7-day culture as described in (**A**) produced in MT2 cells treated with 60 μM SPI-01 or mock treated were normalized by p24 level and then tested for infectivity in uninfected MT2 cells. Infection was detected by p24 levels in the media after 7 days. (**C**) Pseudovirus produced in HeLa cells treated with 60 μM SPI-01 or mock treated were tested for infectivity in different cells. Infection was detected by luciferase activity from HIV encoding luciferase. (**D**) Effect of SPI-01 on activation of latent HIV-1. SPI-01 did not affect activation of latent HIV-1 by TNF-α(left panel). Infectivity of HIV-1 generated from SPI-01-treated latency cells (U1/HIV-1), when activated by TNF-α, is reduced (right panel). (**E**) Viral replication in CD8-depleted PBMCs challenged with the NL4-3 strain of HIV-1 (MOI of 0.01) in the presence or absence (control) of 50 μM SPI-01. Viral replication was assayed based on HIV-1 p24 ELISA on days 4, 7, 10, 12, and 15 post-infection. Error bars represent standard deviation of experiments performed in triplicate.

To examine the effect of SPI-01 on yield of infectious HIV-1, CEM-CCR5 and MT2 cells were treated with 0, 30 or 60 μM SPI-01 for one hour, then stocks of wild-type HIV-1_NL4–3_ and HIV-1_IIIB_ were added to initiate infection. After 16 hours, excess virus and SPI-01 were washed away by replacing cell culture medium in order to examine the infectivity of the second wave of virus produced from cells after the initial infection and in the absence of SPI-01. Cells were cultured for 7 days in media without the inhibitor, and viral levels were assayed by p24 ELISA. The 16-hour SPI-01 treatment dramatically decreased virus production (Fig. 2A). The viruses from the media after 7-day culture, HIV-1_NL4–3_ or HIV-1_IIIB_, were normalized by p24 ELISA, and then used to infect uninfected MT2 cell. The results confirmed that viruses produced under SPI-01 treatment were less infectious that those produced under vehicle control treatment (Fig. 2B). Similar results were obtained with VSV-G pseudotyped virus (Fig. 2C). Although these results suggest that virus packaging was altered, viral protein contents did not appear to be altered (Figure S1). This differs from HIV-1 protease inhibitors, which are also known to affect HIV-1 infectivity by preventing maturation of the viral proteins and alter virion morphology^17^,^18^. Additionally, we investigated the infectivity of virus produced by reactivation of infectious HIV-1 in latent cellular models J-Lat 10.6, 8.4 and 9.2, as well as U1/HIV-1 cells^19^ (Fig. 2E). SPI-01 did not activate viral replication or affect activation (Fig. 2D, left panel). The infectivity of the reactivated virus was quantitatively examined using the 1G5 cell line, a Jurkat derivative that contains a stably integrated HIV-1-LTR-luciferase construct^20^. In comparison to cells treated with TNF-α only, those treated with TNF-α and SPI-01 were significantly less infectious (p < 0.05, Fig. 2D, right panel). As mentioned above, SPI-01 was not toxic to PBMCs at up to 100 μM (Fig. 1F), and at 50 μM it inhibited viral replication in PBMC (Fig. 2E). Taken together, these results indicate that treatment of cells with SPI-01 allowed continued production of virus but yielded virions that had severely reduced infectivity.

### The effects of SPI-01 is on HIV integration in a SUMOylation-dependent manner

**Figure 3:**
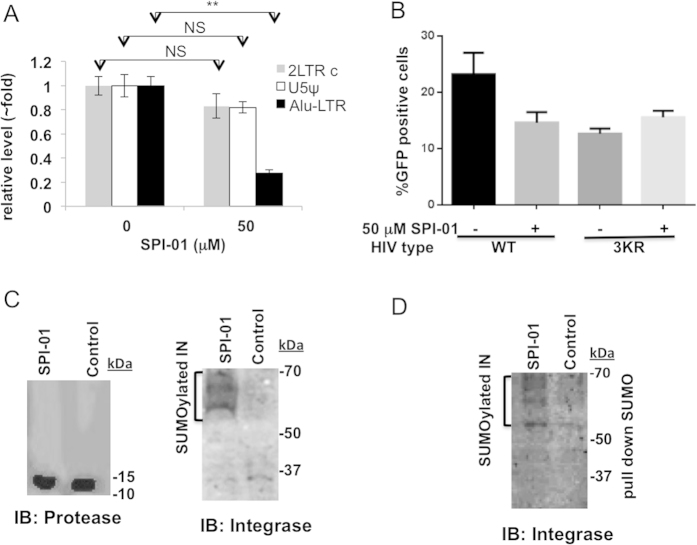
SPI-01 inhibits HIV-1 infectivity is dependent on integrase SUMOylation. (**A**) qPCR analysis of late reverse transcription product (U5ψ) and 2-LTR circles in Hela cells that were infected for 5 h with DNAse-treated virus supernatant generated from 293T cells treated with 60 μM SPI-01 or mock treated. qPCR was also used to analyze the integration product (Alu-LTR) after infection for 24 h. NS denotes p > 0.05 and “**” denotes p < 0.01 from statistical analysis with student t-test. (**B**) The effect of SPI-01 (50 μM) on infectivity of pseudotyped pNL43 containing a wild type integrase (WT) or SUMOylation defective integrase (3KR). (**C**) Western blot analysis of integrase and protease in VSV-G-pseudotyped pNL43LUCR^-^E^-^ virus from transfected 293T cells generated in the presence of SPI-01. Input was normalized by p24. Additional bands identified in integrase produced under SPI-01-treatment are indicated by bracket. (**D**) Western blot analysis of SUMOylated integrase by pull-down of SUMOylated proteins from viral lysates followed by immunoblotting with an anti-integrase antibody. Cells were treated with 20 μM SPI-01 or mock treated. Same amounts of virus inputs from SPI-01-treated and mock-treated cells were used as determined by p24 level.

To further understand the defect of the virus produced by SPI-01 treated cells, we measured the preintegrative viral DNA in cells infected with virus produced from cells treated with SPI-01 or vehicle. The amounts of late reverse transcription product (U5ψ) and 2-LTR circles^21^,^22^,^23^ amplified by qPCR appeared to be not statistically significantly different either in cells that were infected with virus produced from SPI-01-treated cells or from mock-treated cells (Fig. 3A). This indicates that the defect in infectivity was not in cell entry nor was it caused by lack of viral RNA or defect in reverse transcription activity. In addition, the detection of 2-LTR circles indicates that translocation of the reverse transcriptase (RT) product to the nucleus was not inhibited^23^. However, using Alu-based qPCR^15^, we observed that virus produced from SPI-01-treated cells had significantly lower integration efficiency than virus from mock-treated cells (Fig. 3A).

Because SPI-01 was found to inhibit SENPs and because it is shown that integrase-specific SUMO modification is critical to viral infectivity^24^, we investigated whether the loss of integration activity of virus is linked to SUMO modification of HIV-1 integrase. To investigate this, we used a plasmid (pNL4–3EnvFsGFP) that encodes infectious HIV-1 but with a mutant integrase that has all three SUMOylation sites in the integrase sequence mutated from Lys to Arg (HIV-1_3KR_). 293T cells treated with SPI-01 or vehicle control were transfected with the same amounts of VSV-G and pNL4–3EnvFsGFP encoding either a wild type HIV-1 (WT, control) or SUMOylation defective integrase containing HIV-1 (HIV-1_3KR_). The same amounts of VSV-G-pseudotyped virions, HIV-1_WT_ (SPI-01 or control-treated) and HIV-1_3KR_ (SPI-01 or control-treated), as determined by p24^CA^ ELISA, were used to infect 293T cells. Infectivity was tested by the percentage of GFP-positive cells. While the infectivity of the wild type HIV-1_WT_ virus was reduced by SPI-01, the infectivity of the mutant virus HIV-1_3KR_ was not (Fig. 3B). This result suggests that the effect of SPI-01 is mechanistically linked to integrase SUMOylation.

To investigate the status of integrase in the virus that lost infectivity, we performed western blot analysis of p24-normalized and pelleted virus produced from SPI-01 or mock treated 293T cells. Incorporation of the HIV-1 protease into virions was not affected by SPI-01 treatment (Fig. 3C). When blotting for integrase, it was found that virus produced in the presence of SPI-01 showed additional bands above the 50 kDa marker that was not seen in the mock-treated control, but was similar in size to that previously reported for SUMO-modified HIV-1 integrase (Fig. 3C)^24^. This suggested that SPI-01 inhibited the removal of SUMO moieties from the SUMOylated integrase before viral packaging and resulted in incorporation of modified integrase into the virus. To confirm that the additional band in HIV-1 integrase blot was SUMOylated integrase, 293T cells were co-transfected with a plasmid mixture containing plasmids encoding His_6_-tagged-SUMO-1, -2 and -3 together with plasmids encoding VSV-G and pNL43LUCR^-^E^-^, and were mock treated or treated with SPI-01. His_6_-tagged-SUMOs allowed the pull-down of SUMOylated proteins under denaturing conditions to eliminate non-covalent protein-protein interactions and to preserve SUMOylated proteins during sample preparation and pull-down. Viruses produced were pelleted and lysed. SUMO-modified proteins were then pulled down by Ni-NTA beads, followed by immunoblotting with an anti-integrase antibody. Again, similar bands above the 50 kDa marker only appeared in viruses produced from SPI-01-treated cells (Fig. 3D), suggesting SUMOylation of the integrase.

## Discussion

In this study, we have demonstrated that a family of previously identified HIV inhibitors does not inhibit HIV entry, transcription and production of new virons, but the virons produced from cells treated with these compounds are defective in integration. However, these viral particles are capable of entry, have full RT activity and the resulting viral cDNA can enter into the nuclease. This mode of action against HIV infectivity is distinct from that of the well-characterized small molecule inhibitors of infectivity – HIV protease inhibitors. HIV protease inhibitors also inhibit the production of infectious virons, but these virions lack RT activity^18^.

Our data suggests that the defect in HIV-1 integrase activity of the virus produced from cells treated with SPI-01 is related to integrase SUMO modifications. It has been found recently that the HIV-1 integrase is a SUMOylation substrate^24^. SUMO modification of the HIV-1 integrase did not affect its enzymatic activity nor did it affect integrase nuclear localization. However HIV-1 virions carrying a SUMOylation-defective integrase mutant produced less integration product but their entry and reverse transcription steps were not affected^24^. SPI-01 that inhibits SENPs did not have an effect on the infectivity of virus harboring SUMOylation-defective integrase, but inhibited the infectivity of virus harboring the WT integrase. Therefore, the effect of SPI-01 is dependent of integrase SUMO modification. This finding is consistent with recent whole-genome screens, in which proteins involved in SUMOylation were identified as host factors that affect HIV-1 replication, including SENP5^25^,^26^.

To our knowledge, mutant integrase genes with modified SUMOylation sites have not been reported. This is likely because any mutant virus that escapes the inhibitor treatment is unlikely to emerge, and if it does emerge, would have diminished replication fitness and, hence, significantly reduced infectivity compared to wild-type virus. Consistently, we could not generate HIV-1 resistance to SPI-01 over an 8-week exposure *in vitro* (data not shown) using the same protocol that generated AZT and Raltegravir-resistant virus in two weeks^27^,^28^. This result further supports that SPI-01 targets a host factor and not a viral factor, because mutation in the host genome would be required for such resistance.

The findings described here could contribute to strategies for curing HIV-1. HIV-1 infection remains incurable due to the latently infected CD4^+^ T cells. The current “shock and kill” strategies for a cure of HIV-1 involve activation of HIV-1 from latency, e.g. using histone-deacetylase (HDAC) inhibitors^29^,^30^,^31^, and then HIV-1 antigen-expressing cells would be destroyed, e.g. using immune T cells. Such approaches are in early stage development in human trials, but have limited success^32^,^33^,^34^. An inhibitor of virus infectivity could significantly improve the success of such a strategy, by significantly suppressing the infectivity of free virus generated by reactivation. SPI-01 represents a small molecule class and strategy to inhibit HIV infectivity, and inhibition of SENPs may contribute a new strategy for cure of HIV-1 by inhibiting the production of infectious virions upon activation from latency.

## Methods

### Cells culture conditions

Jurkat, U1/HIV-1, MT2 and CEM-CCr5 cells (AIDS Research and Reference Reagent Program, National Institute of Allergy and Infectious Diseases) were cultured in RPMI 1640 medium supplemented with 10% FBS, 100 units/ml penicillin (RPMI-10), 100 μg/ml streptomycin, 0.2 mM L-glutamine, and 20 mM HEPES. 293T human embryonic kidney cells and HeLa cells were cultured in complete DMEM plus 10% FBS, 100 units/ml penicillin, 100 μg/ml streptomycin, and 0.2 M L-glutamine.

### Toxicity assay

Cells (CEM, MT2; 20,000 cells/100 μl) were seeded in each well of a 96-well plate. Serial dilutions of SPI-01 were added in triplicate to wells. Control wells received the same volume of medium only. Cells were incubated (4 days, 37 °C), after which the numbers of live and dead cells were counted as follows. Cells (50 μl) were transferred to a well of a 96-well plate and mixed with trypan blue (50 μl), followed by counting the numbers of live and dead cells. The cytotoxic cut-off was determined as the average percentage of live cells in the control wells.

CD8-depleted human PBMC cells were treated with SPI-01 at 0, 25, 50 or 100 μM concentrations in triplicate. 5 × 10^4^ un-infected cells (total 95 ul/well) were placed into a 96 well plate, maintained in RPMI + 30 U/mL IL-2), and challenged with HIV-1 (NL4–3) at MOl of 0.01. Cells were collected on Day 12 and washed with phosphate buffer saline. Then 0.25 μL of dye of the Live/Dead® Fixable Dead Cell Stain Kits (Life technologies) were added to the cells and mixed well. The dye was allowed to incubate with the cells for 30 minutes in the dark. Then the cells were washed and fixed with 400 μL of 4% formaldehyde for 15 minutes. The percentage of live and dead cells was determined by flow cytometry.

### Generation of single cycle reporter virus and transduction

293T cells were transfected with a reporter plasmid pNL43LUCR^-^E^-^ and a plasmid expressing VSV-G envelope protein (a gift from Dr. Gary Whittaker, Cornell University) using lipofectamine plus (Invitrogen). After incubation (6 h), the transfection media was replaced with complete media containing 0.2% DMSO (control) or 60 μM SPI-01. After 48 h, supernatants were collected and filtered through a 0.45-μm disk, and virus was normalized by p24 ELISA of the supernatant. Target cells were infected with virus for 5 h before media was replaced. Luciferase analysis (Promega luciferase assay kit) was carried out after 48 h of infection. All infectivity assays were carried out in triplicate.

### Quantitative real-time PCR for provirus DNA detection

Proviral DNA was quantified as described before^35^ and VSV-G-pseudo-typed pNL43LUCR^-^E^-^ was used to infect cells. Briefly, cells were collected after 5 hours of infection for late reverse transcription product (U5ψ) and 2-LTR circles, and 24 hours of infection for integrated provirus (Alu-LTR) detection, respectively. Total cellular DNA was extracted using the QiaAmp DNA-extraction kit (QIAmp DNA blood minikit; Qiagen, Madrid, Spain), following the manufacturer’s protocol. Quantitative amplification of provirus DNA was performed using the following primers and probes (MH531, 5′-TGTGTGCCCGTCTGTTGTGT-3′; MH532, 5′-GAGTCCTGCGTCGAGAGATC-3′; MH535, 5′-AACTAGGGAACCCACTGCTTAAG-3′; SB704, 5′-TGCTGGGATTACAGGCGTGAG-3′; SS-F4, 5′-TGGTTAGACCAGATCTGAGCCT-3; LTR-R5, 5′- GTGAATTAGCCCTTCCAGTACTGC-3′; LRT-P, 5′- CAGTGGCGCCCGAACAGGGA-3′ and P-HUS-SS1, 5′-TAGTGTGTGCCCGTCTGTTGTGTGAC-3′). The probes were labeled with the fluorophore 6-carboxyfluorescein [FAM] and the Black Hole Quenche1. To normalize provirus DNA per cell, amplification of the cellular 18 S gene was performed using the TaqMan 18 S control reagents kit (Applied Biosystems, Roche, Barcelona, Spain). The PCR was performed in a total volume of 20 μl using 1 × TaqMan Universal PCR master mix (Applied Biosystems), a 0.6 μM concentration of the primers, 0.08 μM of the probes, and 0.45 μg of the DNA sample. Reactions were analyzed with the Applied Biosystems 7900HT Real-Time PCR system using SDS 2.4 software (Applied Biosystems). For control sample, a parallel heat-inactivated virus was used to infect the cells in a separated dish as well in order to eliminate carryover contamination from plasmid DNA.

### Virus characterization

The protein content in virions was confirmed by polyethylene glycol (PEG) concentration, centrifugation, and western blot analysis. After p24 ELISA normalization, filtered virus supernatant was mixed with a final concentration of 10% PEG in an eppendorf tube. The mixture was centrifuged (30 min, 4,000 rpm, 4 °C), and the pellet was re-dissolved in SDS-PAGE sample loading buffer containing 100 mM dithiothreitol (DTT) and immunoblotted for viral components and SUMOylation profiles.

### Quantitative activation of latent HIV-1

J-Lat cell lines 10.6, 8.4 and 9.2 or U1/HIV-1 were treated (2 h) with DMSO (control) or 60 μM SPI-01. Then the latency cells were stimulated with either 10 ng/ml TNF-α (Sigma), or 5 μM Prostratin. After 24 h, the J-Lat 10.6 cells were fixed with 4% formaldehyde, and flow cytometry analysis was performed to assess GFP expression. For U1/HIV-1 cells, 24 h after activation, cells were co-cultured with 1 G5 cells for 48 h. Then, the cells were lysed and analyzed for luciferase activity.

### Virus with SUMOylation-defective integrase

HEK293T cells seeded at a confluence of 80% (2 × 106 cells) were transfected with 1 μg pNL4–3EnvFsGFP (WT) or pNL4–3EnvFsGFP (3KR) and 0.3 μg pCMV-VSVG using Lipofectamine 2000 according to manufacturer’s instructions. After a six-hour incubation, the transfection solution was replaced with fresh media containing either DMSO or the SPI-01 at a concentration of 50 μM. The culture was incubated for an additional 40 hours and the virus was harvested from the culture supernatant for the p24 assay. HEK293T cells were infected with the virus equivalent to 20 ng of p24 and the fraction of GFP + cells was determined by FACS 48 hours after infection.

### Detection of integrase SUMOylation

293 T cells were transfected with a combination of the following plasmids: 10 μg pNL4–3, 6 μg VSV-G and 8 μg each of His-SUMO-1, -2 and -3 using calcium phosphate precipitation method or Lipofectamine® LTX with Plus™ (Life Technologies) in each 10 cm dish. Then the media were changed to complete media containing 20 μM SPI-01 or 0.2% DMSO (control). 48 h later, supernatants were collected, filtered and HIV pseudoviruses were normalized by p24 enzyme-linked immuno assay (ELISA) and concentrated with PEG8000. The same amounts of virions produced under SPI-01 or mock treatment were lysed under denaturing conditions in a buffer A (6 M guanidinium–HCl, 0.1 M Na_2_HPO_4_/NaH_2_PO_4_, 0.01 M Tris–Cl pH 8.0, 5 mM imidazole, 10 mM β‐mecaptoethanol). The lysates were incubated with Ni^2+^‐NTA beads (Qiagen) for 4 h at room temperature. Then beads were washed with buffer A followed by buffer B (8 M urea, 0.1 M Na_2_PO_4_/NaH_2_PO_4_, 0.01 M Tris–Cl pH 8.0, 10 mM β‐mercaptoethanol) and then buffer C (8 M urea, 0.1 M Na_2_PO_4_/NaH_2_PO_4_, 0.01 M Tris–Cl pH 6.3, 10 mM β‐mecaptoethanol). The bound proteins were eluted with buffer D (200 mM imidazole, 0.15 M Tris–Cl pH6.7, 30% glycerol, 0.72 M β‐mercaptoethanol, 5% SDS), subjected to SDS-PAGE, and were detected by Western blot for HIV integrase with an anti-integrase (aa 1–16) antibody (HIV AIDS reagent program). Viral lysates without Ni^2+^-NTA pull down were also directly loaded to SDS-PAGE and detected by the same anti-Intergase antibody by Western blotting.

### Infectivity assay with peripheral blood mononuclear cells

Peripheral blood mononuclear cells (PBMCs) were obtained from healthy donors at City of Hope National Medical Center using discarded anonymous blood unit leukocyte filters (Pall). PBMCs were eluted and then isolated by centrifugation through a Ficoll–Hypaque solution (Histopaque-1077; Sigma). CD8 + T-cells (cytotoxic T cells) were depleted with CD8 Dynabeads (Invitrogen) according to the manufacturer’s instructions. CD8-depleted PBMCs were washed twice in phosphate-buffered saline, resuspended in TCM 1000 ActiCyte media (BioE) for activation of two days then maintained in RPMI + 30 U/mL IL-2 (5% CO_2_, 37 °C). CD8-depleted PBMCs (50,000 cells) were challenged with the NL4-3 strain of HIV-1 at a multiplicity of infection of 0.01 in the presence or absence of 50 μM SPI-01, and culture supernatants were collected 4, 7, 10, 12, and 15 days post-infection. Viral replication was assayed on these days using HIV-1 p24 ELISA (Perkin Elmer) according to the manufacturer’s instructions.

## Additional Information

**How to cite this article**: Madu, I. G. *et al.* A Novel Class of HIV-1 Antiviral Agents Targeting HIV via a SUMOylation-Dependent Mechanism. *Sci. Rep.* **5**, 17808; doi: 10.1038/srep17808 (2015).

## Supplementary Material

Supplementary Information
