# Supplementary material for: A Novel Class of HIV-1 Antiviral Agents Targeting HIV via a SUMOylation-Dependent Mechanism
Source: Sci Rep. 2015 Dec 8;5:17808. doi: 10.1038/srep17808 (PMC4672295; doi:10.1038/srep17808)
Supplement: Supplementary Information [file srep17808-s1.pdf]

Supporting Figures

A Novel Class of HIV-1 Antiviral Agents Targeting HIV via a SUMOylation-Dependent Mechanism

Ikenna G. Madu[1], Shirley Li[2], Baozong Li[1], Haitang Li[3], Tammy Chang[2], Yi-Jia Li[1], Ramir Vega[1], John Rossi[3], Jiing-Kuan Yee[2], John Zaia[2], and Yuan Chen[1,*]

[1]Department of Molecular Medicine, [2]Department of Virology, and [3]Department of Molecular and Cellular Biology, Beckman Research Institute of the City of Hope, 1500 East Duarte Road, Duarte, CA 91010.

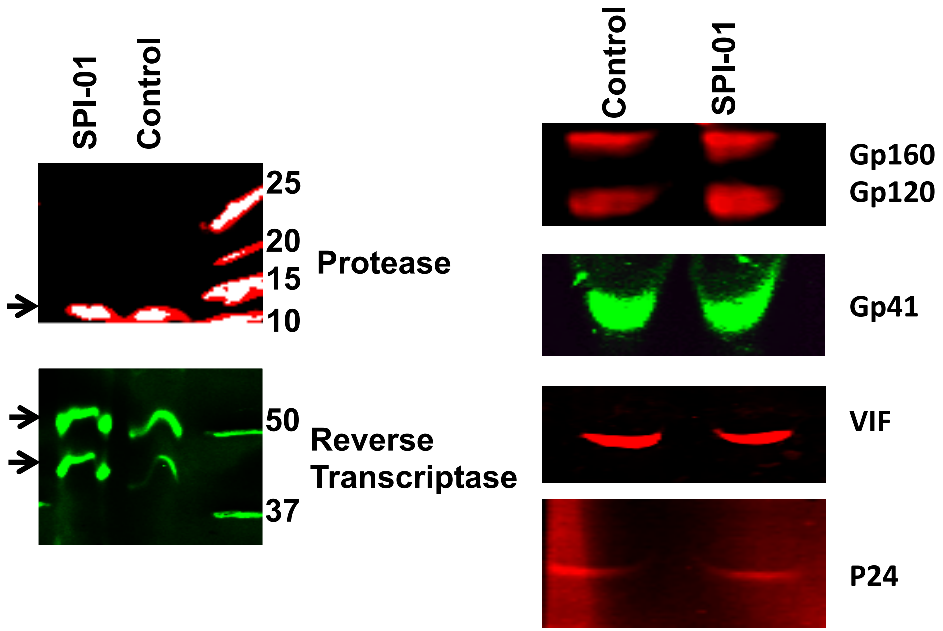

Figure S1. Comparison of viral proteins generated from cells treated with 60 μM SPI-01 or mock treated. VSV-G-pseudotyped single cycle reporter virus were collected from cell media, precipitated by PEG and normalized by p24 levels before Western blot analysis with antibodies against different HIV proteins using an Odyssey imager.

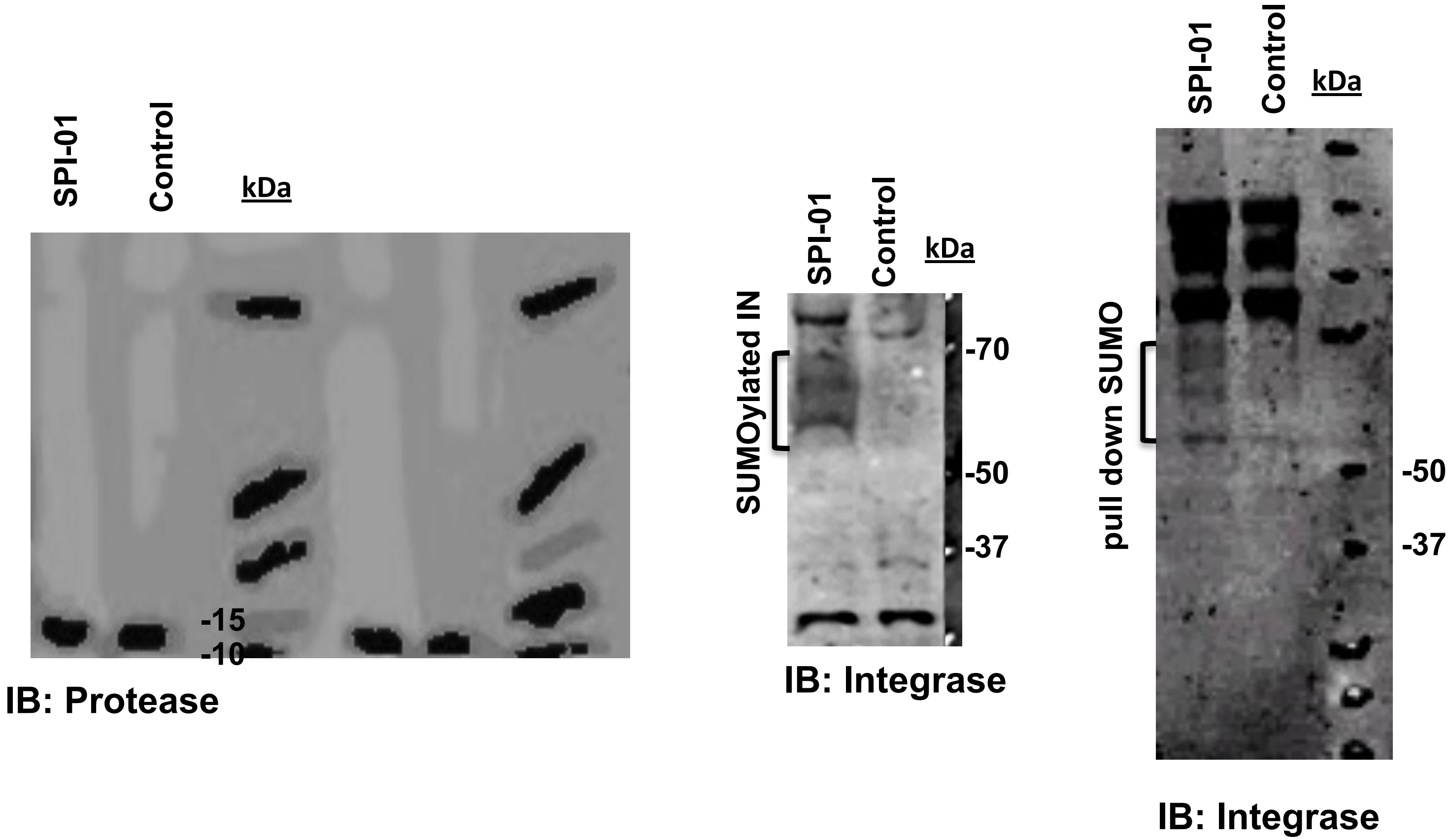

Figure S2. Original uncrop gel images in Fig. 3
